# Regulatory RNAs Involved in Bacterial Antibiotic Resistance

**DOI:** 10.1371/journal.ppat.1004299

**Published:** 2014-08-28

**Authors:** David Lalaouna, Alex Eyraud, Svetlana Chabelskaya, Brice Felden, Eric Massé

**Affiliations:** 1 Department of Biochemistry, RNA group, University of Sherbrooke, Sherbrooke, Quebec, Canada; 2 Université de Rennes, Inserm U835 Biochimie Pharmaceutique, Rennes, France; The University of North Carolina at Chapel Hill, United States of America

## What Are Small Regulatory RNAs?

**Figure 1:**
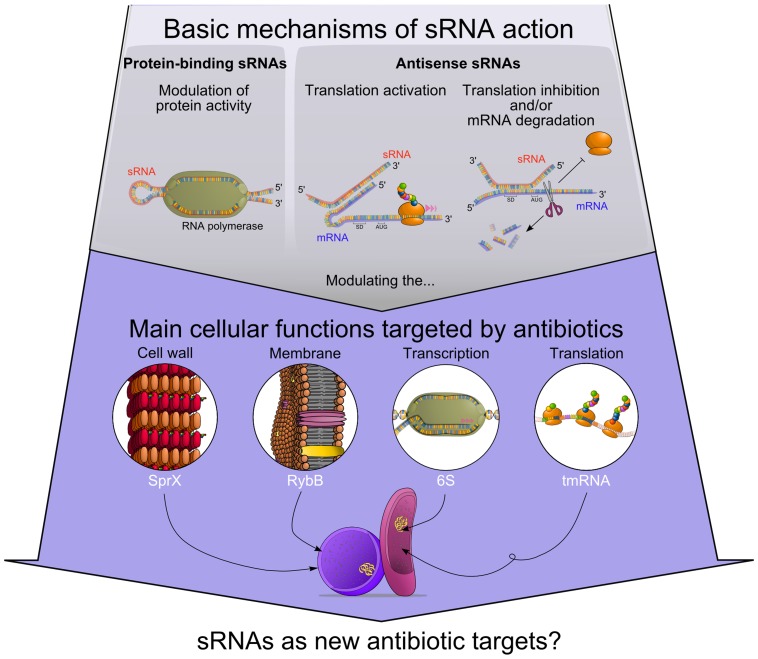
Functional connections between antibiotics' modes of action and regulatory RNAs. sRNAs modulate protein activity or influence target mRNAs fate. Some of those sRNA targets are part of cellular processes that can be affected by antibiotics. Accumulating evidence suggests that several sRNAs alter antibiotic resistance by modulating bacterial cell wall (SprX), cell membrane (RybB), transcription (6S RNA), and translation (tmRNA).

An increasing number of RNAs have been recently shown to possess regulatory functions similar to those of proteins. In bacteria, these regulatory RNAs are usually noncoding and are short size (50–500 nts) transcripts that are often referred to as small RNAs (sRNAs) [1], [2]. These sRNAs are synthesized under specific environmental conditions and play a major role in the regulation of various cellular processes (Figure 1) [3]. Most of them act via an imperfect antisense base-pairing with their target mRNAs. Duplex formation usually results in inhibition or stimulation of mRNA target translation. In some cases, sRNAs can also bind proteins to influence their activities (e.g., 6S RNA). Compared to protein-dependent mechanisms, sRNAs require less energy, act faster and also allow a coordinated regulation of several targets. Owing to these characteristics, sRNAs allow efficient adaptation of bacteria to their ever-changing environment. Therefore, the possibility exists that some sRNAs may be involved in antibiotic resistance. In this report, we provide evidence that illustrates the growing number of sRNAs that influence bacterial resistance to antibiotics.

## Is There a Link between Antibiotic Exposure and sRNAs Expression in Bacteria?

In the last few years, several studies have shown a correlation between bacterial sRNA expression and antibiotic exposure. An initial study identified four sRNAs in *Salmonella* Typhimurium with an enhanced expression when the bacterium was challenged with antibiotics such as tigecycline or tetracycline [4], both of which inhibit bacterial protein synthesis. Remarkably, deletion of one of these sRNA genes that encodes SroA reduced *Salmonella* Typhimurium viability when exposed to tigecycline. Therefore, SroA sRNA activation in a strain exposed to tigecycline may influence bacterial resistance to that antibiotic. In an additional study using the *Staphylococcus aureus* strain as a model, the expression of some sRNAs correlated with exposure to four antibiotics (vancomycin, linezolid, ceftobiprole, and tigecycline) [5]. These recent data that suggest functional links between sRNA expression and the presence of antibiotics raise the important question whether sRNAs play a role in antibiotic resistance.

## How Can sRNA Functions Lead to Altered Antibiotic Resistance?

### Mechanisms of antibiotic resistance

Antibiotics can use miscellaneous mechanisms of action to target vital processes such as nucleic acid and protein synthesis, as well as envelope integrity. Strikingly, various bacterial sRNAs are involved in these functions (Figure 1) [3]. As a consequence, perturbation of sRNA activity may lead to alteration of cellular processes, and a potential outcome is modulation of bacterial antibiotic resistance.

### sRNAs involved in RNA synthesis

Some antibiotics alter the transcriptional machinery, and sRNAs have been found to modulate this process. For instance, the 6S RNA functions through interaction with housekeeping forms of RNA polymerase holoenzyme in gram-negative and gram-positive bacteria. Even if 6S sRNA has not been shown to directly affect antibiotic resistance by regulating transcription, cells lacking 6S RNA are defective for persistence [6]. Since persistence is a process that makes bacteria highly tolerant to different antibiotics, data suggest a functional link between 6S RNA expression and antibiotic sensitivity.

### sRNAs involved in protein synthesis

Protein synthesis involves translation of an mRNA. Many antibiotics interfere with protein synthesis, targeting various steps of the translation process [7]. Ribosomes stalled by protein synthesis inhibitors (e.g., chloramphenicol) can be rescued by a quality-control mechanism that involves the transfer-messenger RNA (tmRNA). However, cells lacking tmRNA are more sensitive to these inhibitors due to the defects in mRNA and protein quality monitoring [8]. In addition, mutations in tmRNA decrease persister cell survival, thus increasing susceptibility to a variety of antibiotics.

### sRNAs involved in cell membrane integrity

Many antibiotics have to penetrate the bacterial cell envelope to be effective. For instance, the outer membrane of gram-negative bacteria functions as a selective barrier by combining a hydrophobic lipid bilayer with pore-forming proteins (porins). Therefore, any factor influencing bacterial membrane synthesis or permeability could lead to antibiotic resistance. There are two cell-penetrating pathways that can be used by antibiotics: the lipid-mediated pathway for macrolides (e.g., erythromycin) and other hydrophobic antibiotics, and the general diffusion porins for hydrophilic antibiotics (e.g., the family of β-lactams, gentamycin, and kanamycin) [9].

Lipopolysaccharides (LPS) are components of the outer membrane of gram-negative bacteria and provide a barrier against antibiotics using the lipid-mediated pathway [9]. The PhoP/PhoQ two-component system can activate many genes involved in LPS modifications and, by extent, can control resistance to antibiotics [10]. Interestingly, two sRNAs (MicA and GcvB) have been described as regulators of *phoPQ* mRNA in *Escherichia coli* [11]. Moreover, another sRNA, ArcZ, has been shown to directly regulate the expression of a phosphoethanolamine transferase, also involved in LPS modifications [12].

Alteration of outer membrane composition, particularly outer membrane proteins (Omp), represents one of the major mechanisms for antibiotic resistance. Thus, sRNAs such as MicF or MicC, which target the major porins OmpF and OmpC respectively, are likely to mediate antibiotic resistance. Moreover, at least six other sRNAs (InvR, MicA, OmrA/B, RseX, and RybB) have already been described as regulator of Omps [13], thus modulating outer membrane fluidity and permeability in gram-negative bacteria.

### sRNAs involved in the regulation of membrane transporters

The active pumping of antibiotics out of the cells by efflux pump systems also contributes to antibiotic resistance [14]. As an example, the MtrCDE efflux pump and the inner membrane protein MtrF allow *Neisseria gonorrhoea* to resist various hydrophobic antimicrobials, including penicillin and erythromycin. Interestingly, NrrF sRNA reduces *mtrF* mRNA levels and therefore inhibits MtrF production [15].

One additional example is the overexpression of DsrA sRNA, which was found to induce multidrug resistance in *E. coli* [16]. In fact, through its positive control of the alternative sigma factor σ^S^ (or RpoS), DsrA triggers the expression of genes encoding the MdtEF multidrug efflux pump and thus affects antibiotic susceptibility. Since other sRNAs (ArcZ, RprA, and OxyS) are involved in *rpoS* regulation [17], these additional regulators can play a role in the control of the MdtEF efflux pump.

Similarly, the sRNA RyhB activates the translation of *cirA* mRNA that encodes a receptor and translocator of the antibiotic colicin Ia [18]–[20]. Under normal growth conditions, the Shine-Dalgarno (SD) sequence of *cirA* mRNA is not accessible for translation initiation. However, under iron starvation conditions, RyhB sRNA is expressed and pairs with *cirA* and that allows the accessibility to the SD sequence [18]. Consequently, the higher number of CirA transporter produced confers an increased sensitivity to colicin Ia. These results support the interpretation that RyhB modulates the sensitivity to colicin Ia through CirA.

### sRNAs involved in cell wall turnover

Microbial cell wall is an essential structural component that is responsible for reproductive division and for maintaining cellular integrity. Furthermore, the cell wall limits entry of potentially harmful macromolecules into the cell. In a large scale analysis of *S. aureus*, it was observed that a sRNA was down-regulated when cells were challenged with vancomycin and ceftobiprole, two antibiotics that target the cell wall [5]. Remarkably, this down-regulated sRNA was antisense to the *mecA* gene encoding the penicillin-binding protein 2a (PBP2a) which induces a higher level of resistance to β-lactams. This observation suggests a putative role of this sRNA in antibiotic resistance. Recently, another *S. aureus* sRNA, SprX (also known as RsaOR), was shown to inhibit SpoVG protein synthesis, a protein encoded by the *yabJ-spoVG* operon involved in glycopeptide and oxacillin resistance [21].

## Could sRNAs Be Used as Working Models to Design New Antibiotics?

RNAs represent an unexploited area of potential molecules for antibacterial design. The recent discovery of sRNAs as a general class of powerful regulators has revolutionized our understanding of gene regulation. sRNAs are involved in many cellular processes in response to stress or when cells are challenged with antibiotics. Unraveling the functions of sRNAs in virulence and host immunity will provide fundamental knowledge that can be used to develop next-generation antibiotics using sRNAs as original targets. However, many unanswered questions remain with respect to RNA biology, but we are convinced that sRNA-based therapeutic treatment of infectious diseases may become useful tools in the near future.
